# Microtubules in Polyomavirus Infection

**DOI:** 10.3390/v12010121

**Published:** 2020-01-18

**Authors:** Lenka Horníková, Kateřina Bruštíková, Jitka Forstová

**Affiliations:** Department of Genetics and Microbiology, Faculty of Science, Charles University, BIOCEV, 25250 Vestec, Czech Republic; horniko1@natur.cuni.cz (L.H.); katerina.podolska@natur.cuni.cz (K.B.)

**Keywords:** virus, microtubules, polyomavirus, T antigens, VP1 capsid protein, molecular motors, dynein, kinesin, virus trafficking, cell cycle block

## Abstract

Microtubules, part of the cytoskeleton, are indispensable for intracellular movement, cell division, and maintaining cell shape and polarity. In addition, microtubules play an important role in viral infection. In this review, we summarize the role of the microtubules’ network during polyomavirus infection. Polyomaviruses usurp microtubules and their motors to travel via early and late acidic endosomes to the endoplasmic reticulum. As shown for SV40, kinesin-1 and microtubules are engaged in the release of partially disassembled virus from the endoplasmic reticulum to the cytosol, and dynein apparently assists in the further disassembly of virions prior to their translocation to the cell nucleus—the place of their replication. Polyomavirus gene products affect the regulation of microtubule dynamics. Early T antigens destabilize microtubules and cause aberrant mitosis. The role of these activities in tumorigenesis has been documented. However, its importance for productive infection remains elusive. On the other hand, in the late phase of infection, the major capsid protein, VP1, of the mouse polyomavirus, counteracts T-antigen-induced destabilization. It physically binds microtubules and stabilizes them. The interaction results in the G2/M block of the cell cycle and prolonged S phase, which is apparently required for successful completion of the viral replication cycle.

## 1. Introduction

Microtubules are one of three major components of the cytoskeleton of eukaryotic cells. They are indispensable for the intracellular transport of organelles, secretory vesicles, and macromolecular complexes. Further, microtubules are required for a variety of functions, including maintenance of cell structure and polarity, separating chromosomes during cell division, and the beating of cilia and flagella.

Microtubules are cylindrical filaments of varying lengths with an outer diameter of 25 nm. The basic structural unit of a microtubule is a heterodimer of two closely related globular proteins, α-tubulin, and β-tubulin. The heterodimers self-associate using noncovalent longitudinal and lateral interactions and form a stiff, hallow structure build from 13 parallel protofilaments [1]. Microtubules are polarized filaments; the heterodimers self-assemble in a head-to-tail manner creating two distinct ends. Whereas, α-tubulin subunits are exposed on the minus end, β-tubulin subunits are exposed on the plus end of the microtubule [2]. The plus ends of microtubules exhibit rapid dynamics and are responsible for interactions with different cellular structures. In contrast, the minus ends of microtubules grow very slowly and determine the geometry of microtubular networks. Microtubule minus ends are usually stably anchored to their nucleation sites—so-called, microtubule-organizing centers (MTOCs) or centrosomes—and are localized in close proximity to the nucleus [3,4]. Both, α-tubulin and β-tubulin subunits have a binding site for one molecule of GTP and work as enzymes catalyzing GTP hydrolysis after the incorporation of tubulin dimers into the filaments. Only the GTP-bound form of tubulin is capable of the efficient incorporation into the elongating microtubules. In the microtubule lattice, only GTP bound to the β tubulin can be hydrolyzed, and then the GDP remains caught in the filament structure. If the addition of new subunits proceeds more slowly than nucleotide hydrolysis, the microtubules begin to shrink in an event called catastrophe. Microtubules are highly dynamic structures and frequently grow and shrink at a rapid yet constant rate. The continuous transition between the microtubules’ growth and shrinking state is called dynamic instability [5].

The dynamics of microtubules can be regulated by various microtubule-associated proteins (MAPs), and vice versa, tubulin may modulate the activity of its binding partners [6]. Specific MAPs are ATP-dependent microtubule motor proteins, kinesins, and dyneins, which ensure continuous trafficking along microtubules. The energy derived from repeated cycles of ATP hydrolysis is used for the physical walking of microtubule motor proteins along the filaments. Whereas, dyneins typically move along the microtubules toward the minus-end, the majority of kinesins are responsible for movement toward the plus-end of microtubules [7,8,9].

The diversity of microtubule functions within the cell is regulated by the presence of diverse tubulin isotypes and by various posttranslational modifications of tubulin. Tubulin posttranslational modifications, such as polyglutamylation, glycylation, detyrosination, or acetylation of the lysine-40 of α-tubulin residue, regulate microtubule function. Posttranslational modifications of the microtubule act either directly, by modulating their mechanical properties and stability as acetylation, or indirectly by regulating their interactions with MAPs [10]. The microtubule dynamics may be influenced by microtubule destabilizing agents, such as nocodazole, colchicine, or colcemid, or by microtubule-stabilizing drugs, such as taxol or paclitaxel [11,12].

Since microtubules are an indispensable structure for the maintenance of many cell functions, viruses exploit and affect them to ensure the transport of virions, viral proteins, and host proteins necessary for virus propagation into or from the places of virus replication. Moreover, viruses use their gene products to modulate microtubule dynamics for their own benefit.

## 2. Involvement of Microtubules in Polyomavirus Infection

Polyomaviruses (PyVs) are a group of small (~45 nm), non-enveloped, tumorigenic DNA viruses replicating in the nucleus. Their circular dsDNA genome (~5.5 kbp), associated with cell histones (apart from histone 1), is located within the icosahedral capsid. Currently, the *Polyomaviridae* family is divided into four genera comprising 80 species that infect animals, including humans (reviewed in [13,14,15]). The most studied PyVs are model viruses, such as mouse polyomavirus (MPyV) and Simian vacuolating virus 40 (SV40), and human polyomaviruses associated with pathologies, BK virus (BKPyV), JC virus (JCPyV), and Merkel cell polyomavirus (MCPyV). The genomes of polyomaviruses may be divided into early, late, and control regions. The non-coding control region contains an origin of replication and promoter–enhancer elements and plays a role in the regulation of the virus life cycle by controlling its replication and transcription. Transcription proceeds from the control region in both directions—the region of early genes is transcribed from one DNA strand and region-encoding late capsid proteins from the opposite strand. From the early transcript, mRNAs for the early proteins, so-called tumorigenic or T antigens, are created by alternative splicing immediately after infection. The large T antigen (LT) (~80 kDa) and small T antigen (ST) (~20 kDa) are encoded by all polyomaviruses. However, from the early coding region of PyVs, additional splice variants of the early mRNA can be generated for the synthesis of additional gene products. The best-characterized is the middle T antigen (MT) (~55 kDa), a membrane-anchored protein encoded by rodent polyomaviruses, as, e.g., MPyV. T antigens are indispensable for the regulation of viral transcription, and for viral genome replication; they are responsible for dysregulation of the cell cycle of infected cells and for their transforming potential. The late region is expressed after virus replication and encodes three structural proteins: the major capsid protein, VP1, and the minor capsid proteins, VP2 and its shorter variant, VP3 (reviewed in [13,16,17]). VP1 is able to self-assemble into capsid-like particles and non-specifically binds DNA (reviewed in [18]). Moreover, it is responsible for the recognition of surface receptors during virus entry into the host cell (reviewed in [19]). Intact capsids of polyomaviruses are composed of 72 ring-shaped capsomers, made up of five molecules of VP1 associated with one molecule of either VP2 or VP3. The minor capsid proteins are located in the inner site of the central cavity of the VP1 pentamer [20]. In addition to their structural function, minor proteins are indispensable for virus infectivity [21]. Some polyomaviruses express additional virus-specific regulatory proteins. Late in their viral life cycle, SV40-late mRNA encodes a protein called VP4, which is suggested to contribute to the lytic release of virions from the host cell [22]. The late coding region of BKPyV, JCPyV, and SV40 encode a small, mainly cytoplasmatic regulatory phosphoprotein, agnoprotein, which is necessary for the successful finishing of the virus life cycle [17]. In addition to proteins, some PyVs also express microRNAs, which target early viral mRNA sequences and also some cellular transcripts. These viral miRNAs are likely to help PyV evade the host immune system and, by targeting LT, reduce their own replication [23].

**Figure 1:**
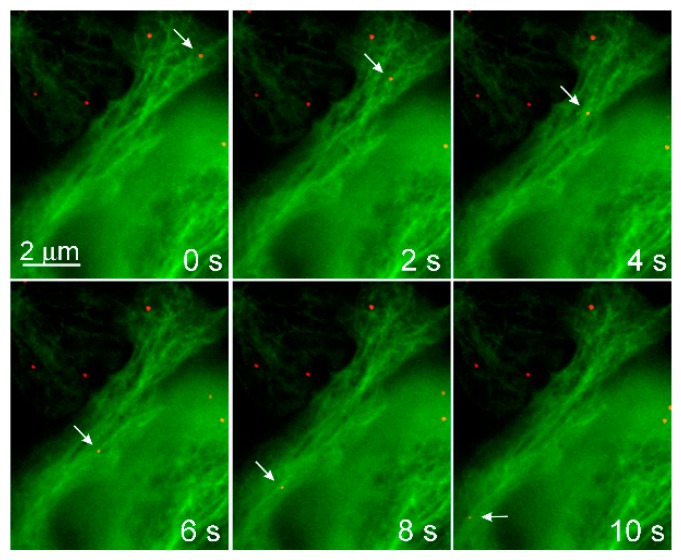
Mouse polyomavirus moves along microtubules. 3T6 cells expressing tubulin fused with enhanced green fluorescent protein (green) were infected with Alexa Fluor 546-labeled mouse polyomavirus (MPyV) (red). Cells were scanned with ΔT = 2 s. Arrowheads point to MPyV virion. Cells were examined with an Olympus IX81 CellR microscope equipped with an MT20 illumination system.

In general, the polyomavirus’ life cycle begins with the interaction of VP1 with a receptor on the cell surface and virions internalization by the host cell. After the endosomal transport of virions through the cytoplasm and delivery of a viral genome to the cell nucleus, T antigens are transcribed. After translation of a sufficient amount of T antigens, viral genome replication by host cell machinery starts, followed by the transcription of late genes, production of structural proteins, virion morphogenesis, and virus release from cells. Microtubules are applied in several stages of the polyomavirus life cycle. Polyomaviruses exploit microtubules for the intracellular movement of viral particles (Figure 1). Moreover, their gene products are able to affect microtubule dynamics and thus modulate cellular function.

### 2.1. Microtubules in Transport of Virions in the Host Cell

#### 2.1.1. Polyomavirus Trafficking from the Host Cell Membrane to the Nucleus.

Polyomaviruses need to travel throughout the cytoplasm to deliver their genomes to their place of replication—the cell nucleus. They enter host cells via endocytosis, and, on their productive pathway, they travel in endocytic vesicles to the endoplasmic reticulum (ER). From the ER, they, partially disassembled, translocate to the cytosol to reach the nucleus via nuclear pores.

Different PyVs recognize different receptors on the host cell surface, despite their virion similarities. Several polyomaviruses studied, including MPyV, JCPyV, BKPyV, and MCPyV, require sialylated glycans for infectious entry into host cells [24]. Different gangliosides have been identified as major receptors for BKPyV (GD1b and GT1b), MPyV (GD1a and GT1b), and SV40 (GM1) [25,26]. Furthermore, additional-cell surface interactions of polyomaviruses have been described—MPyV with α4β1 integrin [27,28], BKPyV with N-linked glycoprotein containing α(2,3)-linked sialic acid [29], or SV40 with class I major histocompatibility proteins [30]. The serotonergic receptors 5-HT2AR, 5-HT2BR, and 5-HT2CR serve as receptors for JCPyV in glial cells [31,32,33], while the initial attachment of JCPyV to the cell surface is mediated by interaction with the receptor motif, α2,6-linked glycan lactoseries tetrasaccharide c (LSTc) [19]. MCPyV binds sulfated glycosaminoglycans (GAGs) for initial attachment. However, it also requires interaction with carbohydrates containing a linear sialic acid motif, such as other PyVs [34]. 

While MPyV [35,36], SV40 [37], BKPyV [38,39], or MCPyV [34] enter cells in monopinocytic vesicles derived from lipid rafts, including caveolae, JCPyV is internalized into clathrin-coated pits [40]. The trafficking of polyomaviruses has been intensively studied on SV40 and MPyV model viruses, and also on human BKPyV, JCPyV, and MCPyV. Although endosomal traveling to the ER differs in detail among individual polyomaviruses, it has many common features. PyV virions do not escape the endosomal system until they reach the lumen of the smooth endoplasmic reticulum. Although the productive trafficking pathway of SV40 [38,41,42], and also MPyV, [35] was first described as independent of low pH of endosomes, later, all studied polyomaviruses were shown to be sensitive to the elevation in endosomal pH [43,44,45,46,47,48]. On their productive endocytic pathway, monopinocytic vesicles with internalized PyVs fuse with early endosomes and later appear in late acidic endosome compartments, from which they are sorted into ER. A dominant-negative mutant of Rab7 GTPase, connected with late endosomes, negatively affected both MPyV and SV40 infection [46,49]. Rab GTPases serve as regulators of membrane transport through their recruitment of effector proteins [50]. The means of PyV transport from late endosomal compartments, and its sorting to the ER, remains obscure. SV40 was shown to utilize COP1 vesicle-mediated retrograde transport [51]. Several studies presented experiments showing that PyVs avoid Golgi complex during their trafficking [47,51]. Some studies suggest that ganglioside receptors GD1a for MPyV or GM1 for SV40 can promote targeting of the virus from late endosomes to the ER [46]. Using whole-genome RNA interference screening, BKPyV was found to exploit Rab18 GTPase activity for trafficking from the late endosomes to the ER [52].

The endosomal virus traveling to the ER, followed by the release of virions to the cytosol for their translocation to the cell nucleus, seems to be pathway-specific for polyomaviruses. Once PyV virions reach the ER, they become partially disassembled by the reduction and isomerization of disulfide bonds of the VP1 major capsid protein performed by redox enzymes, PDI (protein disulfide isomerases). For MPyV, isomerases ERp57, PDI, and ER29 were found to be required [53,54], while for BK and SV40 polyomaviruses, ERdj5, PDI, and ERp57 were found to mediate conformational changes of virions [55,56]. These conformational changes in the ER lead to alterations in the capsid structure and the exposure of the minor capsid proteins, VP2 and VP3, possessing viroporin activity [57,58]. VP2 and VP3 hydrophobic domains are able to bind and perforate the ER membrane [59,60]. For the exit of SV40 virions from ER to cytosol, the remodeling of the ER membrane, leading to the creation of penetration sites (foci), has been observed [61]. Using an siRNA-mediated screen, the luminal chaperones BiP and BAP31, both involved in the endoplasmic reticulum-associated protein degradation (ERAD) pathway, were identified as being essential for infection. They co-localized in discrete foci and promoted the ER-to-cytosol dislocation of virus particles [61]. Other chaperons associated with the ER membrane were identified s and involved in the virion translocation process—the EMC1 membrane protein [62], and DNA J proteins B12 and B14 [63]. In addition, cytosolic chaperons SGTA [64] Hsc70, Hsp105 [65], and Bag2 [66] participate in ER foci formation and the translocation of SV40 virion. 

Partially disassembled virions are released from ER bind importins to be transported across the nucleopores into the nucleus. The importin binding of virions is mediated by nuclear localization signals (NLS) of the major VP1 and/or the minor VP2, VP3 capsid proteins [67,68,69,70]. The mechanism of PyV genome translocation through nucleopores, and also the extent of the virion disassembly of the translocation complex, is still not clear. Interestingly, MCPyV’s appearance in the cell nucleus may depend on the mitotic activity of the host cells and nuclear envelope breakdown [48], similar to what has been described for human papillomaviruses [71].

#### 2.1.2. Involvement of Microtubules and Microtubule Motors in PyV Trafficking from Plasma Membrane to Endoplasmic Reticulum 

Intact microtubules are essential for the virion trafficking of all studied PyVs towards the nucleus. Early studies of mouse polyomavirus, using cytoskeleton destroying compounds, revealed the dependence of MPyV trafficking on microtubules [72,73]. 

Gilbert et al. [73] showed that after an initial uptake of MPyV virions, intact but not dynamic microtubules are required for MPyV infectivity. Colcemid, which disrupts microtubules, inhibited the ability of MPyV to reach the nucleus and replicate, while paclitaxel, a microtubule-stabilizing agent preventing microtubule turnover, had no effect on MPyV infection. Microtubules were not required for the uptake of MPyV from the cell surface, as there was no difference in the kinetics or efficiency of escape from neutralization antibodies with or without intact microtubules. On the other hand, disruption of the actin cytoskeletal network by cytochalasin, added prior to infection, enhanced MPyV infectivity in both epithelial cells and fibroblasts [73]. Confocal microscopy of infected cells revealed that the movement of the internalized MPyV particles from the cytoplasm membrane was accompanied by the transient disappearance of actin stress fibers. The most intensive colocalization of MPyV particles with tubulin was observed in areas close to the cell nuclei [36]. The importance of intact microtubules has also been demonstrated for other polyomaviruses. The infection of SV40 was shown to be dependent on intact microtubules, and transport along microtubules was found to be essential for virus delivery to the ER [74]. The study of the role of microtubules and microfilaments during early BKPyV infection showed that the infection of Vero cells is sensitive to nocodazole treatment-induced disassembly of the microtubule network. Furthermore, in agreement with MPyV studies, the suppression of microtubule turnover with the stabilizing agent paclitaxel had no effect on BKPyV infectivity. Selective disassembly of the actin filaments with latrunculin A did not impede BKPyV infection, while the inhibition of microfilament dynamics with jasplakinolide resulted in reduced numbers of viral antigen-positive cells [75]. The need for intact microtubules was also demonstrated during BKPyV infection in primary human renal proximal tubule epithelial (HRPTE) cells, which are natural host cells for BKPyV [76,77]. However, data from Moriyama and Sorokin [76] suggest that the dynamics of microtubules play an important role in the transportation of BKPyV in HRPTE cells, different from the results demonstrated in Vero cells. Surprisingly, the study also suggested, based on inhibition experiments using EHNA (erythro-9-(2-hydroxy-3-nonyl)adenine), an inhibitor of dynein ATPase, that the function of microtubule motor, dynein, is dispensable for BKPyV infection of HRPTE cells [76]. The need for intact microtubules for MCPyV has been revealed recently [48].

The infection of another human polyomavirus, JCPyV, which enters cells by clathrin endocytosis, was shown to be inhibited by the treatment of glial cells by nocodazole, but also by cytochalasin D, suggesting that, besides intact microtubules, the virus also requires actin microfilaments for productive infection [43]. 

The involvement of microtubules and their motors in the productive endocytic trafficking of MPyV was studied in more detail. Time-lapse microscopy of alive cells expressing EGFP-fused tubulin or actin, and infected with fluorescently labeled MPyV virions, revealed that both microtubules and dynamic actin are involved in the motility of vesicles loaded with virus cargo. The vesicles moved along microtubules over a long distance in a bi-directionally manner, being transported into the cell interior and back to the cell periphery [49]. The dynamics of transport reflected the characteristics of microtubular, motor-driven, fast transport with bi-directional saltatory movements [78]. Although the virus cargo was transported in both directions (often along an identical microtubule), the virus fluorescent signal was found later post-infection to accumulate around the nucleus, indicating that MPyV transport to the vicinity of the nucleus is prevalent during a longer time-span of trafficking. The inhibition of dynein mediated movement by the overexpression of dynamitin, preventing the transport of the virus to the ER and inhibiting infection. Overexpression of dominant-negative forms of kinesin-1 and kinesin-2 did not significantly affect virus localization and infectivity [49].

Endosomes containing the virus were also propelled over a relatively short distance by actin polymerization. MPyV-loaded endosomes, associated with assemblies of dynamic actin, recruited at their membranes, moved with a substantially lower speed than those moving along microtubules [49]. The role of dynamic actin in the productive trafficking of SV40 has been confusing. An early study by Pelkmans et al. [74] described that the productive pathway of SV40 leads from caveola-derived vesicles to peripheral caveolin-rich, non-acidic organelles, caveosomes named by the authors. From caveosomes, the virus travels rapidly in membrane vesicles to the ER along microtubules. Following the study by this group, dynamic actin was found to be necessary for the closure of caveolae by membrane fission and for “free passage deeper to the cytoplasm”. They also showed that latrunculin did not prevent infection. The movement from caveolae-derived vesicles to caveosomes remained unclear [79]. Later, the caveosome concept was dismissed, caveosomes were identified as modified late endosomes or lysosomes [80], and SV40 was shown to use classical early-to-late endosomal pathways [47]. Moreover, it has been shown that caveolin-1 is not required for SV40 internalization [81].

The observation that caveola-derived endocytic vesicles carrying SV40 virions are driven by dynamic actin into multicaveolar endosomes was described previously. Engel et al. [47] showed that SV40 internalization and initial transport to early endosomes and immature late endosomes are inhibited by actin-affecting agents, while intact microtubules are required for the maturation of late endosomes and subsequent transport of SV40 virions to the ER [47].

Different results were obtained for MPyV. To unravel the contribution of vesicle transport along microtubules and vesicle trafficking driven by dynamic actin to productive MPyV infection, quantification of virions present in individual endocytic compartments was performed in non-treated cells and cells treated with a microtubule-disrupting agent, nocodazole, and actin polymerization preventing agent, latrunculin [49]. Latrunculin A treatment did not prevent MPyV trafficking to early endosomes, late endosomes, recycling endosomes, or to the ER. Moreover, it enhanced its efficiency [49]. Correspondingly, virus infectivity, in the presence of latrunculin A treated cells, was higher than that in non-treated 3T6 cells, in agreement with the observation of [73]. On the other hand, upon nocodazole treatment, localization of MPyV was significantly perturbed, and the virus was retained at the cell periphery, often within membrane multicaveolar complexes, and, later, post-infection in caveolin-rich compartments. 

During the MPyV studies, it has been observed that large numbers of internalized PyVs are led to a non-productive pathway and fail to deliver their genomes to the nucleus [82]. The dominant-negative mutant of Rab11 GTPase did not affect infection efficiency, suggesting that recycling endosomes are not the destination leading to productive infection [45,49,82]. In addition, dynamic actin, in the presence of a microtubule destroying agent, mediated accumulation of MPyV in peripheral multicaveolar-like complexes. These results, together with the observed increase in MPyV infection upon latrunculin treatment, suggest that movement, mediated by dynamic actin, is dispensable for virus infection; moreover, it decreases the productive trafficking of the MPyV virions, targeting them to nonproductive pathways. Interestingly, an almost complete reversibility of inhibition of virus infection by nocodazole (after its washout) was observed, suggesting that microtubules are required for the maturation of late endosomal compartments and may even mediate the link for MPyV between multicaveolar-like structures and acidic endosomes. Several studies have reported caveolin-1 or caveolae trafficking into early endosomes [38,83], and also into MVBs and endolysosomal compartments [80,84].

#### 2.1.3. The Role of Microtubule Motors and Microtubules in the Release of Virus Particles from the ER and in PyV Genome Translocation to the Cell Nucleus.

The microtubule motor, kinesin-1, was found to be exploited in an SV40-induced formation of “foci” on the ER membranes for the translocation of partially disassembled virions from the ER to the cytosol [85]. The authors revealed that kinesin is recruited to the ER membrane by binding to the transmembrane chaperon, a B14 J-protein cumulated in SV40 foci. They further showed, by cell-based, semi-permeabilized ER-to cytosol transport assay [61], that the expression of the dominant-negative (DN) mutant of kinesin-1 (kinesin family protein, KIF5), but not its wild type form, reduced SV40 VP1 levels in cytosol. The expression of the DN mutant of kinesin did not affect the translocation of the cholera toxin from the ER into the cytosol [85]. This suggested that kinesin-1 specifically mediates the penetration of SV40. Neither kinesin-2 and kinesin-3 nor kinesin-5, promoted focus formation. Nocodazole treatment decreased the formation of foci complexes, suggesting the importance of microtubules in the process. The majority of large ER membrane foci localized with the central region of the microtubule network, strongly stained with antibodies against acetylated microtubules. Moreover, treatment of SV40 infected cells with tubacin (inhibitor of histone deacetylase 6; HDAC6), which increased microtubule acetylation, enhanced membrane foci formation and SV40 infectivity. Furthermore, the treatment of infected cells with taxol after the virus reached the ER increased virus infection [85]. Previously, it has been demonstrated by single-molecule imaging that kinesin-1 motors move preferentially on a subset of microtubules, identified as the stable microtubules marked by post-translational modifications, e.g., acetylation, while kinesin-2 and kinesin-3 do not exhibit any preference in microtubule binding [86]. Thus, the results of Ravindran et al. [85] support the hypothesis that the kinesin-1 motor, together with stable acetylated microtubules, is exploited to create a membrane penetration site for the release of SV40 virions to the cytosol for their further transport to the cell nucleus. Whether the mechanism is also applicable for other polyomaviruses remains to be revealed. Interestingly, in the case of MPyV, the expression of the dominant-negative mutant of kinesin-1 did not decrease infection efficiency [49]. 

Ravindran et al. [87] demonstrated that, upon reaching the cytosol, SV40 virions recruit the cytoplasmic dynein to further disassemble the viral particle [87]. Using an unbiased protein–protein interaction approach, they identified cytoplasmic dynein as a binding partner of cytosol-localized SV40. They showed that dynein is neither important for the formation of SV40-induced foci complexes on the ER membrane, nor for SV40 release into the cytosol, but, based on the timing of dynein inhibition, they suggested that SV40 recruits dynein upon reaching the cytosol from the ER. Authors speculated whether the dynein-mediated viral disassembly leads to a partial exposition of viral genome for more easy passing nucleopore, or dynein-mediated disassembly exposes the NLS (nuclear localisation signal) present in VP2 and VP3 to target the virus to the nucleopore, or both. The unsolved question is whether the disassembly of the virus particles occurs prior to or after its delivery to the nucleopore proximity [87]. Interestingly, the disassembly of adenovirus particle-docking by nucleopore prior to the entry of virus genomes to the cell nucleus is mediated by the kinesin-1 motor [88]. On the other hand, a recent study on another non enveloped virus, Parvovirus B19, pointed to the involvement of dynein in the transport of virus particles released from endosomes into the proximity of the nucleopore and, also, in the induction of conformation changes in virus particles occurring in the cytosol (facilitated by the depletion of capsid-associated divalent cations). The changes in conformation resulted in partial exposure of the genomic DNA prior to particle translocation to the nucleus [89]. 

**Figure 2:**
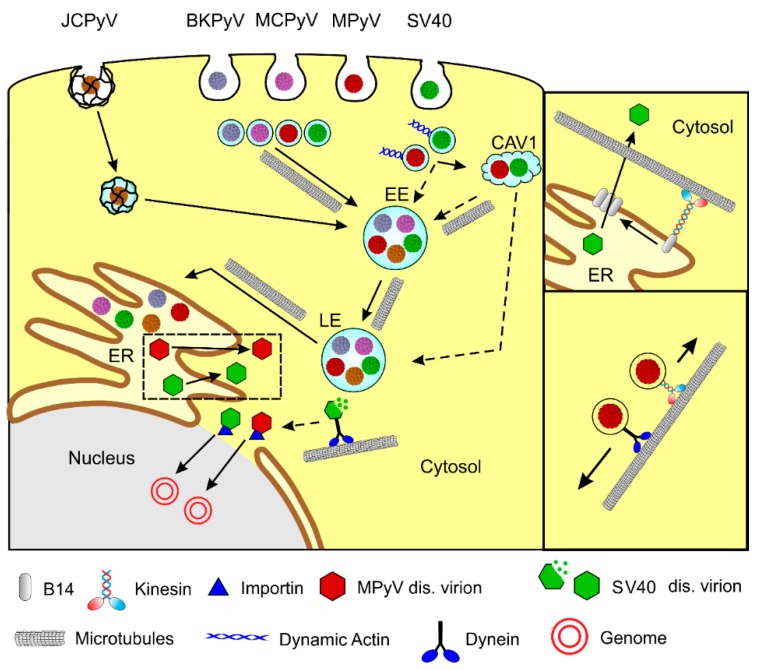
Polyomaviruses utilize microtubules for trafficking towards the cell nucleus. Individual polyomaviruses are, after receptor binding, internalized into monopinocytic vesicles derived from lipid rafts, including the caveolae or by clathrin-coated pits (JC virus). The productive pathway leads through early endosomes (EE), late endosomal compartments (LE) to the endoplasmic reticulum (ER). For the transport along microtubules, polyomaviruses use both dynein and kinesin motors (right lower inset). All polyomaviruses exploit microtubules for their appearance in mature late endosomes and further trafficking to the ER. Dynamic actin propels PyV-carrying vesicles to multicaveolar endosomes (CAV1), representing probably non-productive pathways. For some polyomaviruses, the actin-dependent appearance of virions in early endosomes was suggested. Partially disassembled virions are released from the ER to the cytosol, and virions are consequently further disassembled by dynein. Then disassembled virions translocate, with the help of importins, to the nucleus. Right upper inset: From the ER, the virus is released by the action of cytoplasmic and ER chaperons, including J protein B14 and exposed hydrophobic virus, minor-capsid proteins. The kinesin-1 motor, together with hyperacetylated microtubules, push the B12 chaperon to create large exit foci on the ER membrane. Dashed lines represent hypothetic pathways. MPyV—mouse polyomavirus, SV40—simian vacuolating virus 40, BKPyV—BK virus, JCPyV—JC virus, MCPyV—Merkel cell polyomavirus.

In summary, there are differences in studies regarding the engagement of microtubules and their motors in the traveling of polyomaviruses from the cytoplasmic membrane to the cell nucleus, which may be caused by the nature of the receptors and virion-internalizing endosomes, but also by differences in the cells used for infection. Nevertheless, all the polyomaviruses studied exhibited an indispensable requirement of intact microtubules for the appearance of the virus in late endosomal compartments and virion trafficking to the endoplasmic reticulum. The mechanisms and universality of the engagement of microtubule motors and microtubules in the exit of virions from the ER and their subsequent translocation into the nucleus need to be further studied. The schema of polyomavirus trafficking and the exploitation of microtubules are presented in Figure 2. 

### 2.2. Polyomavirus T Antigens Destabilize Microtubules to Induce Chromosome Instability and Support Tumorigenesis

Although there is almost no evidence characterizing the influence of T antigen expression on the cytoskeleton during infection, there is an increasing number of studies describing the changes in the microtubule network and cytoskeleton in transformed cells or in cells constitutively expressing PyV T antigen(s). Multifunctional T antigens are masters in manipulations of cell function through a broad spectrum of their interaction partners. The target of these manipulations is ensuring and maintaining virus infection. When virus replication is inhibited, T antigens are potent inducers of tumorigenesis, which depends mainly on the ability of LT to bind pRb (retinoblastoma protein) and p53 and thus block their functions (reviewed in [90]). The features of tumor cells include the instability and remodeling of the cytoskeleton, favoring cell movement, and promoting metastasis. Many protein kinases and phosphatases are involved in the regulation of microtubules’ dynamics [91,92,93], which are regulated through the phosphorylation or dephosphorylation of MAPs, including stathmin [94]. In addition, several proteins were identified as tethering the cytoskeleton network together [95]. Hence, the regulation of microtubules by T antigens is achieved by the modulation of microtubule-associated proteins or changes in other cytoskeletal structures, e.g., intermediate filaments. 

The expression of mouse polyomavirus middle T antigen was shown to affect microtubules’ organization [96]. MT interacts with protein phosphatase PP2A [97] composed of three subunits, structural A, regulatory B, and catalytic C subunit [98]. Through this interaction, MT affects the cytoskeleton. In MT-expressing cells, a pool of stable microtubules was depleted, and the association of PP2A with microtubules and PP2A activity was increased. In addition, MT reduced the number of focal adhesions and actin stress fibers, which was accompanied by the redistribution of another MT-interacting protein, SRC protein kinase [96]. The regulation of the microtubule array by MT is caused by the direct effect of PP2A redistribution, as well as indirectly, by the reorganization of the actin cytoskeleton. The microtubule network is further affected by another mouse polyomavirus PP2A-binding protein, the small T antigen. The constitutive expression of ST induced apoptosis in mouse fibroblast cells [99]. In these cells, a subset of microtubules ensuring chromosome alignment during mitosis, kinetochore fibers (k-fibers), were thinner, and the incidence of multipolar spindle formation was increased [100]. Cells were blocked in mitosis, indicating ST interference with mitosis. The k-fibers’ stabilization during mitosis is regulated by the activity of the regulatory subunit B of PP2A [101]. ST interacts with PP2A [97], specifically either with PP2A isoform α or β [102] and by replacing the B subunit in the enzyme complex, thus modulating PP2A function. By replacing the regulative subunit B of PP2A, ST reduces the phosphatase activity of the enzyme. This leads to the destabilization of k-fibers and to aberrant mitosis, which can result in cell death via mitotic-catastrophe associated apoptosis.

The analogous effects of T antigen expression on the microtubule network were described for SV40. Expression of LT antigen promoted the collapse of the intermediate filaments network composed predominantly of vimentin. The breakdown of the vimentin network was accompanied by downregulation of the α-tubulin acetylation and relocalization of stable (acetylated) microtubules to the center of cells [103]. The decrease in microtubule acetylation was a consequence of the stimulation of tubulin deacetylase, HDAC6. These changes in microtubules resulted in an increase in cell stiffness and the promotion of cell invasiveness. LT further negatively affects cell division, and thus induces genomic instability [104,105]. It binds the centrosome-binding protein, the transforming acidic coiled-coil protein 2 (TACC2). This interaction results in spindle instability and aberrant mitosis [106]. Moreover, LT antigen deregulates the mitotic checkpoint, enabling the cell cycle progression despite aberrant mitosis. LT antigen binds the spindle checkpoint kinase, Bub1 [107]. Bub1 delays anaphase progression when kinetochores do not attach chromosomes to microtubule spindles [108,109]. The LT antigen is thus also responsible for preventing spindle assembly checkpoint formation, leading to polyploidy and other genome instabilities. Likewise, MPyV ST, the small T antigen of SV40, triggers mitotic arrest [110]. 

Another polyomavirus, MCPyV, similarly to MPyV or SV40, disrupts microtubules mainly via the ST antigen. Cells expressing T antigens of MCPyV and cells derived from Merkel cell carcinomas exhibit cytoskeletal changes reflected by the increased motility, migration, and invasion of these cells. The ST antigen was shown to be responsible for this phenotype. It has been shown that MCPyV ST expression affects regulatory proteins of the microtubule network, particularly stathmin [111]. Stathmin is a microtubule-associated protein, which regulates microtubule dynamics. An unphosphorylated form of stathmin sequesters free tubulin dimers, thus reducing the substrate for microtubule growth. This ultimately results in microtubules’ destabilization [112]. Knight et al. [111] showed that, in ST-expressing cells, the level of stathmin was upregulated and microtubules were reorganized. The expression of ST resulted in microtubules’ destabilization, which was demonstrated by decreased levels of α-tubulin acetylation, the marker of stable microtubules, and by an increase in unphosphorylated stathmin. Elevated levels of unphosphorylated stathmin were connected with the ST-binding partner PP4C phosphatase, as its dominant-negative mutant abolished ST-induced microtubule destabilization [111]. Moreover, decreased microtubule stability resulted in a defect of cell division. Supernumerary centrosomes, increased aneuploidy, and aberrant mitosis, were observed in cells expressing ST, and increased aneuploidy was observed even in ST-expressing mice [113]. The induction of centrosome instability is connected with the inactivation of the ubiquitin ligase protein complex, SCF^Fbw7^. The loss of Fbw7 function resulted in genome instability and tumorigenesis [114]. The small T antigen interacts with SCF^Fbw7^, abrogating its function. This results in the stabilization of SCF^Fbw7^ targets, for example, LT, c-myc, or cyclin E [115,116]. The accumulation of the SCF^Fbw7^ substrates could be partly responsible for centrosome duplication. All these data revealed the MCPyV ST antigen to be a potent regulator of cytoskeleton dynamics. By the destabilization of the microtubule network, ST regulates cell cycle and promotes host cell genome instability, thereby supporting the tumorigenesis and invasiveness of cancer cells. 

Taken together, these data show that T antigens of polyomaviruses affect the microtubule network by two interconnected mechanisms. Directly, by modifying activities of enzymes regulating microtubules dynamics and indirectly, through the disruption of other cytoskeletal components. These cytoskeletal changes promote cell transformation, as well as the invasiveness of cancer cells and their metastasis. 

### 2.3. Late Proteins Stabilize Microtubules Thus Counteracting T Antigen Microtubule Destabilization 

In contrast to T antigens, the influence of late proteins on microtubules, and the cytoskeleton in general, remains on the edge of interest. It was reported [117] that the JCPyV agnoprotein, the late non-structural protein, associates with microtubules in neuronal cells. The interaction resulted in the dissociation of the fasciculation and elongation protein zeta 1 (FEZ1) from microtubules. FEZ1 is a brain-specific protein, which is involved in several cellular processes, including axon outgrowth, intracellular trafficking, and transcription [118]. The effect of agnoprotein–microtubule interaction on microtubules dynamics has not been described. However, agnoprotein-mediated dissociation of FEZ1 from microtubules inhibited the promotion of neurite outgrowth. Moreover, the overexpression of FEZ1 downregulated JCPyV propagation. In FEZ1 overexpressing cells, the transcription of VP1 and agnoprotein was inhibited, and an active release of virus progeny from the nucleus to the cytosol was suppressed [117]. Therefore, FEZ1 is indicated as a protein with antiviral activity against JCPyV. 

**Figure 3:**
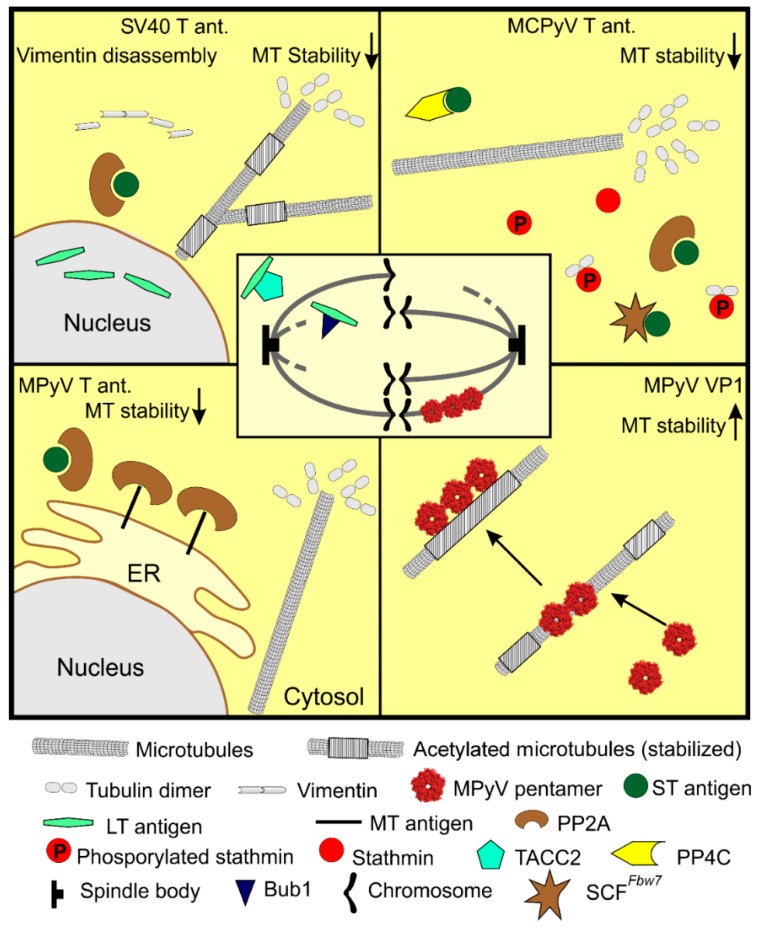
Gene products of polyomaviruses affect microtubule dynamics. Early T antigens affect microtubules indirectly. Small T antigen (ST) and membrane-bound middle T (MT) antigens interact with PP2A phosphatase and alter its redistribution within the cell. The ST antigen is able to replace the regulation unit of PP2A phosphatase and affect its substrate specificity. ST antigen of Merkel cell polyomavirus also binds PP4C phosphatase and SCF^Fbw7^ ubiquitin ligase. As a consequence, microtubule-associated proteins’ (MAPs) functions become deregulated. Mainly, the commutation of dephosphorylated stathmin, which binds tubulin dimers, thus preventing their polymerization, leads to microtubular instability and numeric chromosomal instability. Simian vacuolating virus 40 large T antigen (SV40 LT) disrupts intermediate filaments (vimentin), resulting in microtubule rearrangement. Further, SV40 binds centrosome-binding protein transforming acidic coiled-coil protein 2 (TACC2), and this interaction results in spindle instability. Binding of Bub1 kinase to SV40 LT results in spindle assembly checkpoint failure, resulting in numeric chromosomal instability. In contrast, the late gene product, the VP1 capsid protein of MPyV, binds microtubules directly, causing their stabilization leading to the cell cycle block in G2/M. MPyV—mouse polyomavirus, SV40—simian vacuolating virus 40, MCPyV—Merkel cell polyomavirus.

Several studies describe the direct impact of the major capsid protein, VP1, on microtubules’ dynamics. The studies described the ability of MPyV VP1 to interact with microtubules. MPyV VP1 was shown to interact with microtubules and spindle bodies in early, MPyV-induced epithelial tumors [119]. The authors suggested that VP1-expressing cells may support the development of aneuploidy in tumors. On the other hand, the interference of VP1 with mitosis in tumor cells is toxic as, later in tumor progression, the VP1 gene is silenced [120]. The binding of VP1 to mitotic spindle bodies, followed by mitotic arrest, was further demonstrated in yeast cells. VP1 produced in *Saccharomyces cerevisiae* blocked the colonies’ growth by encasing the mitotic spindle, but surprisingly, after several days, the yeast cells were able to overcome the block by the assembly of a new spindle [121]. The association of VP1 with microtubules and cell cycle arrest was also suggested by Spink and Fluck [122]. They studied MPyV host-range mutants, and one of them did not produce MT and ST antigen but overexpressed VP1. Most of the cells from the infected cell population exhibited the block in the G2/M phase of the cell cycle [122]. Clear evidence of the relationship between VP1-microtubule interaction and blocking of the cell cycle was demonstrated by results from Hornikova et al. [123]. A substantial fraction of VP1 was detected in the cytoplasm, although VP1 possesses a nuclear localization signal, and virus replication and morphogenesis take place in the nucleus. In the cytoplasm, VP1 was found to decorate microtubules. This interaction was supported by the VP1-binding protein, chaperone heat shock protein 90, which helped the assembly of VP1 along microtubules, but did not mediate the VP1–microtubule interaction [123]. Recently, Horníková et al. [124] showed that the level of acetylated α-tubulin, a marker of microtubule stabilization, was elevated in the late phase of infection. In addition, in VP1 positive cells, the level of polymerized microtubules increased, and microtubules that were part of the VP1–microtubule complex were hyperacetylated. Subsequent in vitro experiments revealed that the direct interaction of isolated microtubules and VP1 resulted in microtubule stabilization and a restriction of their dynamics [124]. In agreement with previous studies, VP1 was found to bind the mitotic spindle [119,121,122], and this interaction resulted in a cell cycle block in the G2/M phase [123]. Although the consequence of mitotic spindle stabilization is aberrant mitosis and cell cycle block, the significance of microtubules’ stabilization is not so obvious. The stabilization of microtubules may have several consequences for the infected cell; it is important for intracellular trafficking and for the preservation of cell shape. Since microtubules and other cytoskeletal structures are affected and destabilized by T antigens, in a late phase of infection, VP1 may counteract the T antigens’ destabilization to maintain cell shape and mechanics. The manipulations of microtubules by polyomavirus gene products are summarized in Figure 3.

## 3. Conclusion and Future Directions

Polyomaviruses manipulate and utilize microtubules and/or their motors at all stages of the replication cycle. In particular, they usurp microtubules and their motors towards productive traveling to the nucleus via early endosomes, late acidic endosome compartments, and the endoplasmic reticulum. While several other non-enveloped DNA viruses or capsids of enveloped DNA viruses interact directly with microtubule motors through their capsid protein, polyomaviruses hidden in endosomes exploit the machinery of endocytosis pathways. Endosomes carrying PyV virions travel along microtubules using dynein and kinesin motors. They are also propelled by dynamic actin. While the importance of actin-driven movements for productive PyV trafficking remains questionable, their trafficking along microtubules is essential. Recent studies of events connected with the release of SV40 virions from the ER to the cytosol, and the consequent delivery of viral genomes to the cell nucleus, revealed the exploitation of microtubule motors, not only for classic cargo transport but also for virus removal from the ER or virus uncoating. Polyomavirus early antigens induce destabilization and, as described for SV40, also the rearrangement of the microtubule network. Through interactions with cellular proteins, including kinases and phosphatases, they affect mitosis processes, thus inducing host-genome instability. The petrification of microtubules through their association with the late VP1 capsid protein, observed during the late phase of MPyV infection, resulted in microtubule stabilization and a G2/M blocking of the cell cycle. 

Although the trafficking of polyomaviruses has been studied intensively, many questions remain unanswered. Observed differences occurring in studies of trafficking of different polyomaviruses may be, at least in part, attributed to the diversity of receptors exploited by viruses. In addition, the cell lines used for virus infection may affect virion trafficking. Are there any differences in microtubule and dynamic actin function in the movement in different cell types, e.g., fibroblasts, polarized epithelial cells, or neurons? For MPyV, the movement of virus-carrying vesicles mediated by dynamic actin was shown to be dispensable for productive infection. Moreover, the actin depolymerization agent, latrunculin, increased MPyV infectivity [49,73]. On the other hand, SV40 internalization and virus transport into early endosomes was found to be actin-dependent [47]. The role of actin mediated movement in polyomavirus infection requires further study. 

Studies of MPyV trafficking, performed on cells lacking the ganglioside receptor, revealed that, although the virus binding to the cell surface and its internalization was efficient, the infectivity of the virus was inhibited. After the supplementation of the GD1a receptor to the cells, infectivity was restored [46,125]. Qian et al., [46] demonstrated that the GD1a receptor stimulates not virus binding and entry into cells, but rather, the sorting of MPyV from late endosomes to microtubule-dependent trafficking into the ER. The mechanisms of receptor-dependent polyomavirus sorting directed to the productive pathway is another important subject for future study. 

The discovery that kinesin-1 and microtubules are involved in the release of SV40 virions from the ER to the cytosol, and their suggested mechanisms, should be verified for other polyomaviruses. Furthermore, nothing is known about the trafficking of partially disassembled virions after their release from the ER to nuclear pores. 

A recent study by Ravindran et al. [87] demonstrated that SV40 recruits dynein and mediates further virion disassembly prior to importin-dependent genome delivery into the nucleus. This finding raises questions as to whether microtubules and dynein are involved in the traveling of virions (or released viral genomes) to the nucleopores, what the mechanism of dynein mediated disassembly is, and whether this finding can be generalized to other polyomaviruses. 

Studies on BKPyV revealed that their virion progeny can be actively released from infected cells [126]. There are no data on the role of microtubules and/or other cytoskeleton components in virion trafficking from the nucleus towards the cytoplasmic membrane. How does the virus leave the nucleus? Are nuclear pores involved? Are the virions moved from the nucleus to the plasma membrane naked or in vesicles? Are microtubules important for this movement, and if yes, which motors are involved?

Early antigen-induced destabilization and/or rearrangement of the microtubule network has been studied in cell lines expressing individual T antigens. Thus far, nothing is known about the action of viral gene products in concert with infection conditions. The contributions of these T antigen activities to cell tumorigenesis and invasiveness have been demonstrated. However, their importance for the virus replication cycle remains obscure. Nevertheless, several questions remain to be elucidated in tumorigenesis studies. An interesting observation is that the SV40 LT antigen overriding the spindle assembly checkpoint by interaction with Bub1 kinase is somehow connected with the LT antigen-induced DNA damage response [127]. The T antigens of polyomaviruses modulate DNA damage responses by signaling via two closely related kinases, ATM (Ataxia telangiectasia mutated)/ATR (Ataxia telangiectasia and Rad3 related) to ensure viral genome replication [128,129,130,131,132]. Hein et al. [127] also showed that Bub1 is involved in LT-bound p53 stabilization. Further studies focused on revealing the mechanisms of (i) LT-induced numeric chromosome instability via Bub1, (ii) possible Bub1 functions in the LT-induced DNA damage response, and in LT- mediated p53 stabilization, could gain insight into the relationships among LT actions leading to tumorigenesis and their possible synergistic effect. 

The petrification of microtubules by VP1 binding and the consequent arrest of cells in G2/M was demonstrated for MPyV. Can this be generalized to other polyomaviruses? Does VP1 have any role in carcinogenesis? The binding of VP1 to mitotic microtubules might affect chromosome segregation, leading to chromosome number instability. On the other hand, the expression of VP1 detected in early tumors later becomes silenced [119,120]. What is the reason for this? These and other questions need to be the subject of further studies.
